# The Efficacy of Consensus Tree Methods for Summarizing Phylogenetic Relationships from a Posterior Sample of Trees Estimated from Morphological Data

**DOI:** 10.1093/sysbio/syx086

**Published:** 2017-11-02

**Authors:** Joseph E O’Reilly, Philip C J Donoghue

**Affiliations:** *School of Earth Sciences, University of Bristol, Life Sciences Building, Tyndall Avenue, Bristol BS8 1TQ, UK*

**Keywords:** Bayesian phylogenetics, consensus trees, divergence time estimation, total-evidence dating, morphology

## Abstract

Consensus trees are required to summarize trees obtained through MCMC sampling of a posterior distribution, providing an overview of the distribution of estimated parameters such as topology, branch lengths, and divergence times. Numerous consensus tree construction methods are available, each presenting a different interpretation of the tree sample. The rise of morphological clock and sampled-ancestor methods of divergence time estimation, in which times and topology are coestimated, has increased the popularity of the maximum clade credibility (MCC) consensus tree method. The MCC method assumes that the sampled, fully resolved topology with the highest clade credibility is an adequate summary of the most probable clades, with parameter estimates from compatible sampled trees used to obtain the marginal distributions of parameters such as clade ages and branch lengths. Using both simulated and empirical data, we demonstrate that MCC trees, and trees constructed using the similar maximum *a posteriori* (MAP) method, often include poorly supported and incorrect clades when summarizing diffuse posterior samples of trees. We demonstrate that the paucity of information in morphological data sets contributes to the inability of MCC and MAP trees to accurately summarise of the posterior distribution. Conversely, majority-rule consensus (MRC) trees represent a lower proportion of incorrect nodes when summarizing the same posterior samples of trees. Thus, we advocate the use of MRC trees, in place of MCC or MAP trees, in attempts to summarize the results of Bayesian phylogenetic analyses of morphological data.

Recently developed tip-calibration methods facilitate the inclusion of fossil species and morphological data alongside living species and molecular data for phylogenetic and divergence time estimation, making use of all available relevant data. Indeed, these methods have ignited interest among paleontologists in estimating true evolutionary timescales, even for entirely extinct clades for which only morphological data are available (Bapst et al. 2016; Matzke and Wright 2016; Wright 2017a,b; Wright and Toom 2017). Thus, these methods have the potential to revolutionize our understanding of evolutionary history and unite the hitherto disparate disciplines of paleontological and molecular phylogenetics. However, interpreting the results of such analyses is complicated because morphological data often yield a very diffuse posterior sample of topologically disparate trees that are difficult to reconcile meaningfully in a single consensus tree.

Several methods are available to summarize the results from Bayesian posterior tree samples. A straightforward approach to representing the posterior distribution of trees is to choose a single tree from the sample of trees that can be considered optimal by maximizing some criterion of support. One such approach is to use the single sampled topology with the greatest posterior probability, the maximum *a posteriori* tree (MAP). As Markov chain Monte Carlo (MCMC) sampling is used to obtain a posterior sample of trees it is never certain that the true MAP tree is present in the sample, unless it is possible to obtain an infinite number of samples. This is because the MCMC procedure does not attempt to search for a tree that maximizes the posterior probability but instead samples trees with frequency proportional to their posterior probability. There are $\frac{\left(2n-3 \right)!}{2^{n-2}\left( n-2 \right)!}$ possible strictly bifurcating trees for $n$ taxa (Felsenstein 1978); if the posterior distribution of these trees is very diffuse it is possible that many unique topologies are sampled. To obtain the MAP tree, the MCMC sampling procedure must therefore be performed for an inordinate amount of time as the goal is no longer to approximate the posterior distribution but, instead, to inefficiently find the tree with the greatest posterior probability. Another sampled tree consensus method, Maximum Clade Credibility (MCC), is less susceptible to this source of error as it considers the distribution of clade support in the posterior sample of trees. The MCC method has become one of the most popular consensus methods for summarizing tree samples obtained in tip-calibrated analyses in which morphological data are analyzed (Pyron 2011; Wood et al. 2013; Dembo et al. 2015; Dornburg et al. 2015; Dembo et al. 2016; Herrera and Davalos 2016; Matzke and Wright 2016; Wright 2017a,b; Wright and Toom 2017). This popularity is perhaps because it yields consensus trees that are highly resolved, unlike more conventional methods, such as Majority Rule Consensus (MRC), or alternatively because it is the default method in the popular TreeAnnotator consensus tree construction utility that is often used with BEAST (Bouckaert et al. 2014). The MCC method identifies the single tree in the posterior sample with the largest sum (or alternatively, product) of posterior probabilities across its constituent bifurcations (Heled and Bouckaert 2013). Like the MAP tree, the MCC tree will not explicitly account for topological uncertainty in its structure, as each sampled tree in the posterior sample is invariably fully resolved. As with the MAP tree, the fully resolved nature of the MCC tree is appealing, but it may be a poor summary of the posterior distribution of trees, as it has the potential to include clades with low posterior probabilities that are poorly supported by the data. The potential inclusion of such clades in MCC trees may be caused by morphological data which often yield a diffuse sample of topologically disparate trees from the posterior distribution, principally due to a relative lack of phylogenetic information distributed across a matrix consisting of few characters (Gelman et al. 2013; Steel 2013). Thus, MCC trees based on morphological data, have the potential to include clades with low posterior probabilities which are, by definition, poorly supported by the data and, therefore, likely to be spurious.

The MRC tree method offers an alternative approach in summarizing a distribution of trees by sacrificing the precision associated with potentially erroneous clades for total topological accuracy. MRC trees present divergence times on a set of well-supported (posterior probability $>$0.5) bifurcations, or soft polytomies, in the presence of uncertainty. Such a conservative approach to presenting topological uncertainty may be desirable, particularly in a Bayesian framework in which obtaining the marginal posterior distribution of model parameters results in the explicit estimation of their uncertainty.

For morphological clock analyses, the accuracy of the consensus tree topology upon which clade ages are presented, is integral to the accuracy of the reported timescale. This is because the marginal distributions of clade ages are constructed from only the trees in the sample that are compatible with the consensus topology (Heled and Bouckaert 2013). Reporting ages for spurious clades will have a significant impact on interpretations of evolutionary history. The consensus tree used to summarize the posterior distribution must, therefore, minimize incorrect clades while also maximizing the inclusion of correct clades. Here, using simulated data sets containing variable levels of phylogenetic information, we demonstrate that the increased variance of a finite posterior sample of trees obtained from morphological data is often poorly summarized by MCC and MAP trees as they often include numerous incorrect clades. We also show that MRC trees outperform MCC and MAP trees in summarizing diffuse posterior distributions, presenting a more conservative summary of topology, resulting in the inclusion of fewer incorrect clades. Finally, by analyzing several empirical data matrices that are expected to possess differing amounts of observed information about the same set of divergences, we demonstrate that both the MCC and MAP methods are likely to be inappropriate when summarizing posterior samples of trees obtained from empirical morphological data as consensus trees.

## Materials and Methodology

We simulated matrices that exhibit varying levels of phylogenetic information, performed coestimation of divergence times and topology on these matrices, and then constructed MCC, MAP, and MRC trees from samples of the posterior distribution. To simulate matrices with varying amounts of observed information, we exploited the relationship between the quantity of independent and identically distributed (*i.i.d.*) data drawn from the underlying process in question and the variance of the posterior distribution around the true value of parameter estimates; this relationship is commonly termed consistency (Gelman et al. 2013). We can therefore assume that small matrices simulated with the standard morphological model (Lewis 2001) and analyzed with that same model will produce a more diffuse posterior distribution than larger matrices, and that larger matrices will contain more information about the distribution of parameters in the model.

### Simulated Matrices

All simulations were performed on an arbitrary 36-tip time scaled phylogeny containing four fossil taxa (Supplementary Material available on Dryad at https://doi.org/10.5061/dryad.66s9h). The simulations used the Mk model of morphological evolution (Lewis 2001), with 100 replicate matrices of either 100, 1000, or 10,000 binary characters produced using this model and the assumption of a strict clock.

### Empirical Matrices

We analyzed three empirical matrices spanning common data types used in divergence time estimation. These matrices were obtained by splitting the total-evidence matrix from Ronquist et al. (2012a) into its constituent elements. The first empirical matrix consisted of the morphological characters from this analysis only (353 characters), the second matrix consisted of the molecular characters only (5096 characters), with all fossil taxa removed; the final matrix was a total-evidence combination of both molecular and morphological characters for extinct and extant taxa (5449 characters).

#### Divergence time estimation—

For analyses of our simulated data sets, a posterior sample of trees was obtained using MrBayes 3.2 (Ronquist et al. 2012b). For simulated matrices, errorless point calibrations were applied to noncontemporaneous tips and a root calibration was applied as a gamma distribution with mean $=$ 1 and standard deviation $=$ 0.1. A strict clock was employed with a prior on the rate of $G$(1,2). The Mk model (Lewis 2001) was used to analyze the simulated data. Four chains of Metropolis-coupled MCMC sampling were performed for one million generations, sampling every hundredth generation to produce a final posterior sample of 10,000 trees. For empirical data sets, we performed analyses as in O’Reilly et al. (2015). Consensus trees were constructed for each posterior sample of trees after a 25% burnin, MCC trees were constructed in TreeAnnotator, MAP trees were taken from the MrBayes output files, and MRC trees were constructed by the sumt function in MrBayes.

### Consensus Tree Efficacy Tests

For both simulated and empirical matrices, we performed several tests of the ability of the MCC, MAP, or MRC tree to summarize the posterior distribution of sampled trees. Using a custom R script, we identified all bipartitions present in each individual tree in the posterior sample (post burn-in) for each replicate and then obtained the posterior probability of each of these bifurcations. We use the number of unique bipartitions sampled from the posterior distribution of each analysis to approximate the variance of the posterior distribution itself.

Effective MCMC sampling of the posterior distribution requires a Markov-chain with a stationary distribution that is the same as the posterior distribution, in addition to a finite number of samples that is large enough to accurately approximate the distribution. When MCMC sampling of the posterior distribution is performed, estimated parameter values are sampled with a frequency proportional to their posterior probability. Therefore, if the posterior distribution of a discrete parameter is highly concentrated around the true value of the estimated parameter, a small number of discrete parameter values will be sampled regularly in a finite sample of the posterior distribution. We therefore consider the number of unique sampled bifurcations as an acceptable approximation of the variance of the posterior distribution of topologies.

For simulated matrices, we obtained the number of clades in the MCC, MAP, or MRC tree that are not found in the generating tree, and are therefore incorrect. This was achieved by comparing the constituent taxa of each clade in the generating tree with the constituent taxa of each clade, whether defined by a bifurcation or a soft polytomy, in the consensus trees. If any of the clades in the consensus tree consisted of taxa that did not form a monophyletic clade in the generating tree, they were considered incorrect. We also obtained the number of correct clades within each consensus tree. MCC and MAP trees will possess a constant number of constituent clades, whereas MRC trees may include soft polytomies and will therefore present a variable number of clades. Therefore, we also calculated the proportion of nodes that are incorrect in MRC, MAP, or MCC trees, correcting for the resolution of the consensus tree. For each replicate, we also subtracted the number of incorrect nodes from the number of correct nodes for each consensus tree to obtain a score for the overall accuracy of the estimated topologies.

For empirical matrices we also consider the degree of underrepresentation of clades with relatively high support across consensus tree construction methods. We use three different criteria to determine whether a sampled clade should be represented in the MCC or MAP tree but is missing from either, i.e. if it meets at least one of these criteria it is considered valid but unrepresented. The three separate criteria for a clade to be considered valid but unrepresented are: (i) a posterior probability greater than that of the most poorly supported clade in the MCC or MAP consensus tree; (ii) a posterior probability greater than the mean posterior probability of clades in the MCC or MAP tree; or (iii) a posterior probability greater than 0.5.

## Results

### Simulated Matrices

**Figure 1.**
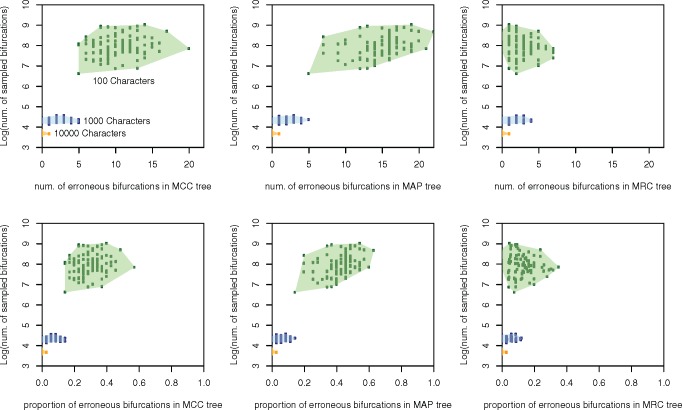
The number and proportion of incorrect bifurcations in MCC, MAP, and MRC summarizations of simulated data sets of different sizes plotted against the number of unique bifurcations sampled in each analysis. MRC trees present fewer incorrect nodes in both absolute and proportional terms, with MCC presenting fewer incorrect nodes than MAP trees. When few characters are analyzed there are more incorrect nodes, in both absolute and proportional terms, in MCC trees than MAP trees. When more characters are analyzed the performance of all three consensus tree methods begins to converge.

**Figure 2.**
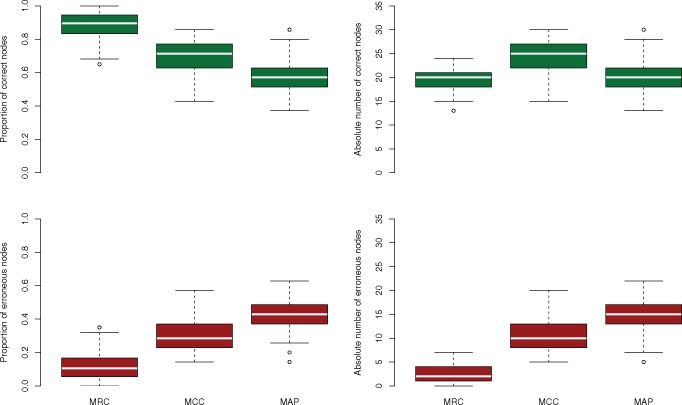
The number and proportion of correct and incorrect clades presented in consensus trees constructed from 100 replicate matrices of 100 simulated characters. MCC trees often present more correct nodes than MRC or MAP trees in absolute terms, but they also present many more incorrect nodes than MRC trees in absolute terms. MRC trees present proportionally more correct nodes and fewer incorrect nodes than either MAP or MCC trees.

**Figure 3.**
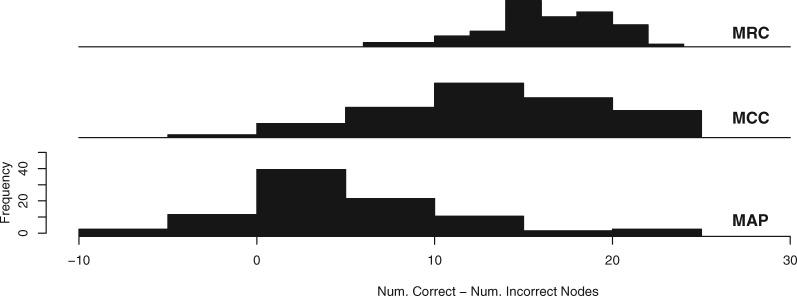
The number of incorrect nodes found within each consensus tree, subtracted from the number of correct nodes found tree within the same consensus trees constructed from 100 replicate matrices of 100 morphological characters. In several cases both MCC and MAP trees contain more incorrect nodes than correct ones.

As the number of simulated characters was increased the posterior sample of trees became more concentrated, as reflected in the decreasing number of unique sampled bifurcations (Table 1). For all consensus tree methods, the mean and range of both the number and percentage of incorrect nodes decreased as the posterior distribution became less diffuse (Table 2). When the posterior sample of trees was at its most diffuse, based on 100-character data sets, MRC consensus trees included far fewer incorrect nodes than MCC or MAP trees, whether expressed in absolute terms or as a proportion of the total number of clades in the consensus tree. MAP trees often contained more incorrect nodes than MCC trees (Table 2; Figs. 1–3). MRC trees recovered from 100-character data sets were never fully resolved, with trees containing a mean of 22 resolved nodes out of a possible 35 (63%), with a range of 16 to 29 (46–83%). MCC trees recovered from 100-character data sets possessed the most correct nodes in absolute terms (on average), with MRC and MAP trees possessing a similar number of correct clades to one another (Fig. 2). Conversely, MCC and MAP trees also included more incorrect nodes than MRC trees in both absolute and proportional terms (Table 2; Fig. 2). When the number of correct nodes is expressed as a proportion of the total number of resolved nodes in the consensus tree, MRC trees greatly outperformed MCC and MAP trees (Table 2; Fig. 2). When the number of incorrect nodes is subtracted from the number of correct nodes presented in each consensus tree, the MRC tree often exhibited a greater total level of accuracy than MCC trees, which in turn often exhibited a higher level of accuracy than MAP trees (Fig. 2). Both MCC and MAP trees occasionally produced topologies with more incorrect nodes than correct nodes, as can be seen in the frequency of replicates with a value below 0 in Figure 3.

**Table 1.** Number of unique sampled bipartitions obtained from the posterior distribution for 100 replicate simulated data sets

	Number of unique sampled bipartitions	
Number of characters	Mean	Range
100	3882.0	1010–9726
1000	208.7	177–246
10,000	176.9	142–220

**Table 2.** Absolute number of incorrect clades in maximum clade credibility (MCC), majority rule consensus (MRC), and maximum *a posteriori* (MAP) trees constructed for posterior distributions sampled from 100 replicate simulated data sets

	MCC		MRC		MAP	
Number of characters	Mean (%)	Range (%)	Mean (%)	Range (%)	Mean (%)	Range (%)
100	10.5 (30.1)	520 (14.3–57.1)	2.7 (11.9)	0–7 (0–35)	14.8 (42.3)	5–22 (14.3–62.9)
1000	1.69 (4.8)	0–5 (0–14.3)	1.03 (3)	0–4 (0–12.1)	1.72 (4.9)	0–5 (0–14.3)
10,000	0.03 (0.1)	0–1 (0–2.9)	0.02 (0.1)	0–1 (0–2.9)	0.03 (0.1)	0–1 (0–2.9)

*Note*: The percentage of nodes that are incorrect for each consensus tree method are presented in parentheses.

The differences between the performance of MRC, MAP, and MCC trees diminished as the number of analyzed characters increased and the posterior sample became less diffuse, with differences between the three methods becoming indistinguishable when 10,000 characters were analyzed (Fig. 1; Table 2; Supplementary Material Figures available on Dryad).

### Empirical Matrices

The posterior sample of trees obtained using molecular data was the least diffuse of all three data types (Table 3). The addition of morphological data in the total-evidence analysis dramatically increased the number of sampled clades and, therefore, yielded a more diffuse posterior sample. It should be noted, however, that this analysis involves more taxa and will therefore naturally allow for more unique sampled bipartitions. Analysis of morphological data on its own produced the most diffuse posterior sample, as inferred from the number of unique sampled clades.

**Table 3.** Features of valid but unrepresented clades in MCC trees constructed from posterior distributions obtained using different data types

Data source	Number of sampled clades	Number of clades missing from MCC tree with pp $>$minimum pp of clades in MCC tree	Max pp of clades missing from MCC tree with pp $>$minimum pp of clades in MCC tree	Number of MRC clades not presented in MCC tree
Molecular	256	13	0.55	1
Morphology	94,660	41,623	0.84	8
Total evidence	29,610	1372	0.78	3

The number of valid but unrepresented clades derived from each set of posterior samples, increased with inferred diffusion, with molecular data resulting in MCC and MAP trees with few overlooked clades and the smallest posterior probabilities for unrepresented clades (Tables 3 and 4). The addition of morphological data, or the analysis of morphological data alone, greatly increased the number of clades unrepresented in MCC and MAP trees (Tables 3 and 4).

**Table 4.** Features of valid but unrepresented clades in MAP trees constructed from posterior distributions obtained using different data types

Data source	Number of clades missing from MAP tree with pp $>$minimum pp of clades in MAP tree	Max pp of clades missing from MAP tree with pp $>$minimum pp of clades in MAP tree	Number of MRC clades not presented in MAP tree
Molecular	31	0.90	5
Morphology	41,629	0.95	13
Total evidence	5803	0.65	4

The taxa that defined the valid but unrepresented clades that possessed the greatest support in each consensus tree (Table 3), were as follows: total-evidence MCC (*Tenthredonoidea, Aglaostigma, Dolerus, Taxonus*); total-evidence MAP (*StephanidaeA, StephanidaeB, Meg*- *alyra, Trigonalidae, Chalcidoidea, Evanioidea, Ichneumo*- *nidae, Cynipoidea, ApoideaA, ApoideaB, ApoideaC, Vespidae, Stephanogaster, Leptephialtites, Cleistogaster, Symphytopterus*); morphology-only MCC (*Sirex, Xeris, Urocerus, Tremex*); morphology-only MAP (*Runaria, Paremphytus*); molecular-only MCC (*StephanidaeA, StephanidaeB, Megalyra, Chalcidoidea, Cynipoidea*); molecular-only MAP (*Paremphytus, Tenthredonoidea, Aglaostigma, Dolerus, Selandria, Strongylogaster, Monop*- *hadnoides, Metallus, Athalia, Taxonus, Hoplocampa, Nematinus, Nematus, Cladius, Monoctenus, Gilpinia, Dip*- *rion, Cimbicinae, Abia, Corynis, Arge, Sterictiphora, Perga, Phylacteophaga, Lophyrotoma, Acordulecera, Decameria).*

## Discussion

Using simulated data, the results of our analyses show that when summarizing a diffuse posterior sample of trees, MAP and MCC consensus trees often include many correct clades, but at the cost of the inclusion of large numbers of incorrect clades. Conversely, MRC trees resolve fewer clades in total, but those that are resolved are less likely to be incorrect. 100-character data sets yield a reduced level of information regarding the distribution of the parameters of the model. In this case, the MRC tree often outperforms the MCC and MAP trees in minimizing the inclusion of both the absolute number and percentage of incorrect clades (Figs. 1 and 3). With larger numbers of simulated characters there is increased information about the estimated model parameters and, therefore, the posterior distribution is expected to become more concentrated around the true value of the model parameters (Gelman et al. 2013). In these cases, MCC, MAP, and MRC methods obtain comparable levels of accuracy, as measured by the proportion of incorrect clades that are presented by these consensus tree methods (Fig. 1). Our results suggest that, when analyzing matrices with large numbers of informative characters, the use of either the MCC, MAP or MRC method is justifiable, but when there is a paucity of information in the observed data, such as when morphological data are analyzed, a more considered choice needs to be made. This choice must be based on whether the inclusion of an incorrect clade is considered worse than the omission of a correct clade; the MRC tree will likely minimize the inclusion of incorrect clades, the MCC tree is more likely to maximize the inclusion of accurate clades, and the MAP tree will include fewer correct clades, and therefore more incorrect clades, than the MCC tree. When choosing consensus tree construction methods, the trade-off between type I and type II errors has previously been explored in a decision theory framework, with the MRC tree considered the optimal topology if the inclusion of an incorrect clade is considered worse than the exclusion of a correct clade (Holder et al. 2008). The results we present here are congruent with this, suggesting that the optimal consensus tree for reporting parameter estimates from morphological data sets is likely to be the MRC tree.

**Figure 4.**
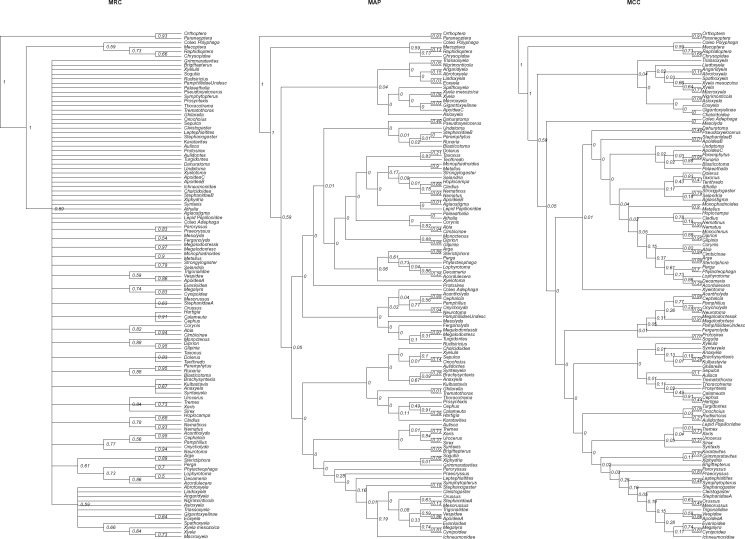
MRC, MAP, and MCC consensus trees for Hymenoptera constructed from a posterior sample of trees obtained when only morphological data was analyzed. The trees are presented as cladograms with arbitrary branch lengths as summary of the posterior distribution of branch lengths results in numerous branches with negative length for the maximum clade credibility tree. The posterior probability of each clade is presented as a node label. In the MCC and MAP trees many clades express very low support and most are not consistent with molecular phylogenies or conventional understanding of Hymenopteran evolution. For the MRC tree few clades can be presented with support $>$0.5, but those that are present are broadly congruent with established Hymenopteran phylogenetic relationships. Posterior probabilities of 0 are induced by the rounding of values to 2 decimal places.

Categorical morphological data can inform parameter estimates alone, or in tandem with molecular data, in a total-evidence approach (Pyron 2011; Ronquist et al. 2012a). By using the number of uniquely sampled bipartitions as a proxy for the variance of the posterior distribution, we have shown that empirical analyses that utilize morphological data in both exclusive and total-evidence approaches, are likely to possess a markedly more diffuse posterior distribution than if molecular data from the same group is analyzed alone, and are therefore less suitable for summary using MCC or MAP trees. Indeed, the MCC tree constructed from the posterior distribution obtained from morphological data alone, contains few clades consistent with other published hymenopteran phylogenies (Fig. 4; Supplementary Material Figures available on Dryad) (Klopfstein et al. 2015; Zhang et al. 2016). Conversely, the MRC tree contains few resolved clades, but those that are presented are broadly congruent with established phylogenetic relationships. The exclusion of correct, well-supported, clades in MCC and MAP trees is particularly worrying as this will influence understanding of both evolutionary relationships and, when divergence times are presented on a consensus tree, our understanding of evolutionary timescales. For example, in the total-evidence MCC tree, the best-supported unrepresented clade has strong support, with a posterior probability of 0.78 (Table 3). These taxa are resolved in a clade in the MCC tree that includes three other taxa, but this has less than 50% support (posterior probability of 0.43), which will not therefore be resolved in the MRC tree. Similarly, the best-supported unrepresented clade in the morphology-only MAP tree has strong support (posterior probability of 0.95; Table 3). However, the smallest clade that contains these taxa also includes an additional taxon and exhibits $<$0.009 posterior probability (Supplementary Material Figures available online on Dryad), effectively precluding the resolution of a strongly supported clade in favor of a very poorly supported clade.

When summarizing a diffuse posterior distribution, the propensity of the MCC and MAP trees to include spurious clades is intimately linked to their inability to represent topological uncertainty as soft polytomies. A finite sample of trees from a highly concentrated posterior distribution will consist of only a small number of clades that have been visited frequently by the MCMC algorithm. In such circumstances, there is a high probability of the set of best-supported clades appearing simultaneously in any sampled tree and, therefore, the MCC or MAP tree. Conversely, when the tree sample is diffuse, the probability of the set of best-supported clades appearing simultaneously in any single sampled tree is diminished, increasing the chances that the MCC or MAP tree will include poorly supported and incorrect clades. This problem is likely to be exacerbated as the number of taxa included in an analysis increases as this will also increase the number of possible bifurcations, reducing the chance of the best set of bifurcations appearing simultaneously. Alternatively, MRC trees represent frequently sampled and, therefore, well-supported, clades exclusively, collapsing poorly supported bifurcations into soft polytomies. Naturally, this reduces the number of spurious and infrequently sampled, poorly supported clades. Our results highlight the fact that poorly supported clades should not be considered credible in general, as collapsing such clades into soft polytomies often improves the overall topological accuracy of a consensus tree.

The relative efficacy of consensus tree construction methods has a large influence over the presentation and interpretation of divergence time estimates in molecular, morphological or total-evidence clock analyses. To obtain the marginal distributions on node ages, the tree sample is examined for clades that are compatible with the consensus tree and a set of node ages is constructed for each clade in the consensus tree from these compatible sampled trees (Heled and Bouckaert 2013). Therefore, it is likely that for morphological clock or total-evidence dating analyses, estimates of clade age are more likely to be presented for incorrect or poorly supported clades in MCC and MAP trees, than in MRC trees. As MCC and MAP trees are more likely to present age estimates for spurious clades, the MRC tree can be considered the optimal topology when inferring evolutionary timescales.

While the presentation of topological uncertainty through the use of soft polytomies often improves the overall accuracy observed in MRC trees when morphological data are analyzed, the potential lack of resolution means that such trees may not be suitable for further downstream analyses that either require a strictly bifurcating topology or erroneously interpret soft polytomies as true multifurcating divergence events. Such downstream analyses should preferably use the posterior sample of trees as input and allow the topological uncertainty of this distribution to be an intrinsic part of the analysis. If this is not possible then the MCC or MAP tree may be considered as potential input trees but, as shown here, these trees are likely to contain poorly supported clades that will impact negatively on the accuracy of estimates that are dependent on these topologies and timescales.

## Conclusion

Using simulated data, we have demonstrated that MCC and MAP consensus trees often include more incorrect nodes than MRC trees when attempting to summarize particularly diffuse posterior distributions. With empirical data, we have shown that when morphological data are added to analyses, MCC and MAP methods have a propensity to include poorly supported and likely incorrect clades. These results suggest that MCC or MAP trees may be unsuitable for use with phylogenetic methods that attempt to integrate morphological data, especially those in which parameter estimates are summarized as annotations on a consensus tree, such as divergence time estimation analyses.

## Supplementary Data

Data available from the Dryad Digital Repository: https://doi.org/10.5061/dryad.66s9h.
